# Enhanced Ultrasound Image Formation With Computationally Efficient Cross-Angular Delay Multiply and Sum Beamforming

**DOI:** 10.1016/j.ultrasmedbio.2025.05.023

**Published:** 2025-06-28

**Authors:** Cameron A.B. Smith, Matthieu Toulemonde, Marcelo Lerendegui, Kai Riemer, Dina Malounda, Peter D. Weinberg, Mikhail G. Shapiro, Meng-Xing Tang

**Affiliations:** a Division of Chemistry and Chemical Engineering, California Institute of Technology, Pasadena, CA, USA; b Department of Bioengineering, Imperial College London, London, UK; c Department of Earth Science and Engineering, Imperial College London, London, UK

**Keywords:** Ultrasound, Acoustics, FMAS, DMAS, CADMAS, Computational efficiency, Ultrafast imaging, B-Mode, AM, Gas vesicles, Contrast

## Abstract

**Objective:**

Ultrasound imaging as a clinical tool is commonly achieved using the delay and sum (DAS) beamforming algorithm, which has limited resolution and suffers from high side lobes. Recently nonlinear processing has proven to be an effective way to enhance image quality. In this work, we describe a new beamforming algorithm called Cross-Angular Delay Multiply and Sum (CADMAS).

**Methods:**

CADMAS takes advantage of nonlinear compounding across planewave steering angles to enhance image contrast. This is then implemented with a mathematical reformulation to produce images at a low computational cost. We tested CADMAS in both conventional B-Mode and amplitude modulation imaging across in vitro and in vivo datasets, and for microbubbles and gas vesicles. We compared the results to DAS and two other nonlinear beamformers, frame multiply and sum (FMAS) and delay multiply and sum (DMAS), as well as the combination of the two.

**Results:**

Our results show on average across our datasets a 7.6 to 40 dB improvement in image contrast over DAS and a 4.8 to 20 dB improvement over DMAS. The computation time of CADMAS is between 1 and 2 times that of DAS; in our implementation we experienced a less than 6% increase in computation time.

**Conclusion:**

Our results show a robust improvement in contrast across multiple datasets both in vitro and in vivo, demonstrating its potential for biological and clinical applications.

## Introduction

Receive Beamforming is a signal processing technique designed to estimate the spatial distribution of a series of sources. It is necessary to produce an image from the signals received by an array of sensors. In the field of medical ultrasound, this is commonly achieved by utilizing the delay and sum (DAS) algorithm [1–3], which attempts to solve this problem by calculating the time of flight from transmission to reception for a set of transducer elements at a specific location, finding the signal received by each element at this time point and then summing these signals before envelope detection. The intensity of the signal is then assigned to the pixel corresponding to that location before repeating the process for all pixels in a desired image. This algorithm, while computationally efficient, has limited resolution and suffers from high side lobes, limiting its effectiveness [4].

One way to improve image quality is to take advantage of adaptive beamforming algorithms, many of which were originally developed for RADAR applications but have since been demonstrated to be effective for medical ultrasound. Most of these algorithms are based on the Capon beamformer [5–7], which applies a data-specific weighting to each element in the beamforming process in an attempt to minimise the contribution of noise and incoherent signals. While these algorithms improve image quality, they significantly increase computational cost.

A more recent set of beamformers use nonlinear processing to improve image quality in a more computationally efficient manner [4, 8–11]. A major contribution was the Delay Multiply And Sum (DMAS) algorithm [4], which cross-multiplies the signals received by different elements by combinatorially coupling elements. This can be interpreted as the aperture auto-correlation function, computing the spatial cross-correlation among the received signals. In doing so, the DMAS algorithm successfully reduces side lobes and improves image quality.

Later, the Frame Multiply And Sum (FMAS) algorithm [8] was developed and applied to ultrafast power Doppler imaging. This algorithm utilises nonlinear angular compounding. It performs conventional DAS on each plane wave angle transmission used in coherent compounding in plane-wave ultrafast imaging and then cross-multiplies pairs of acquisitions to evaluate the spatial coherence in the generated radio frequency (RF) images. This improves the contrast-to-noise ratio over DAS at a computational cost only marginally higher than DAS as the number of angles is usually small.

In this work, we present a new algorithm called Cross-Angular Delay Multiply and Sum (CADMAS), which improves the image quality of ultrafast imaging by evaluating the coherence between all received signals received across all of the elements and across all acquisitions at different angles. This produces enhancements over DMAS which only considers the coherence across elements and FMAS which only considers the coherence across angles. Additionally, we take advantage of a mathematical reformulation, decreasing the computation time compared to conventional implementations of DMAS, to create a beamformer that enhances image quality at a low computational cost.

## Materials and methods

### CADMAS algorithm

**Figure 1:**
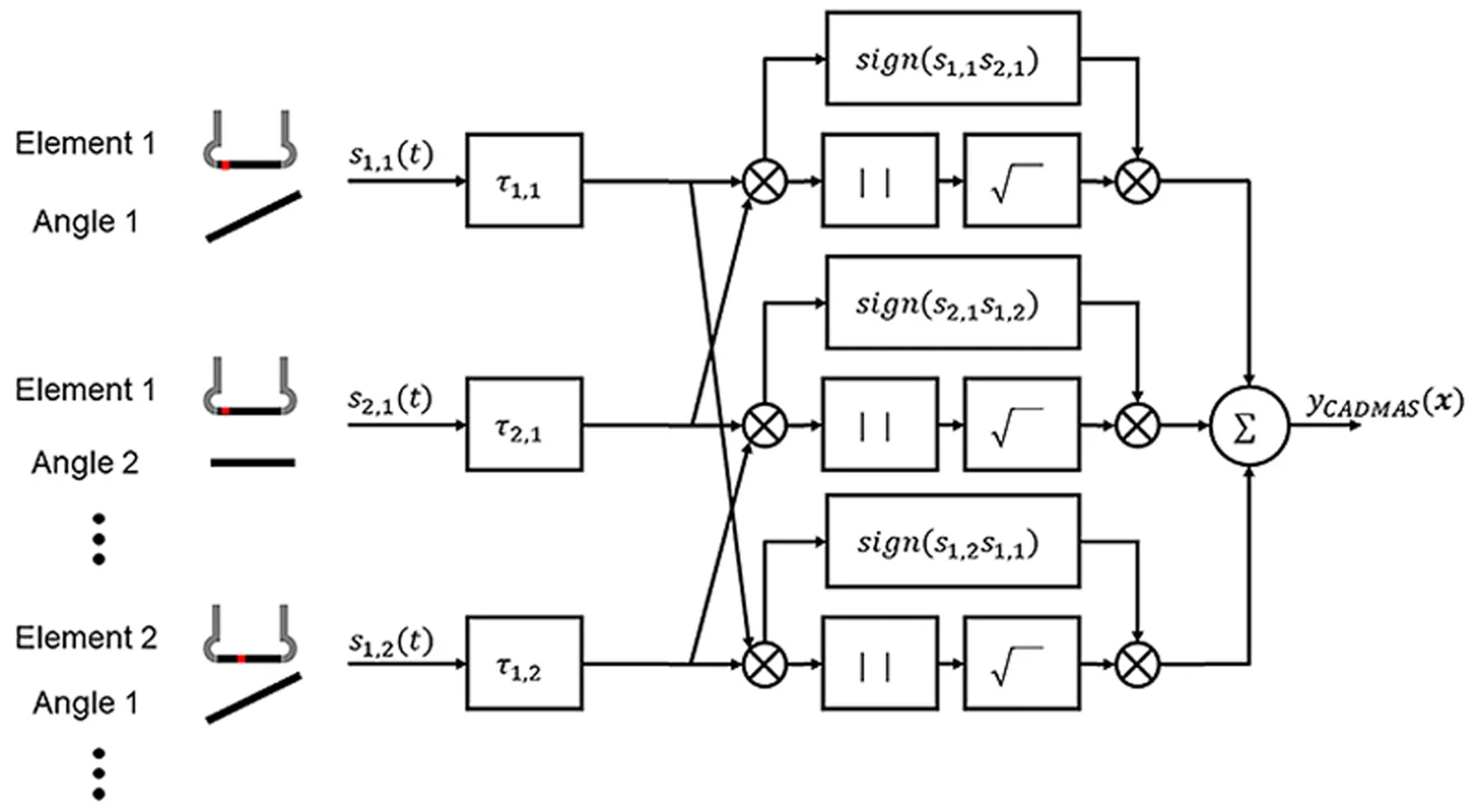
Block diagram showing how the CADMAS algorithm cross multiplies recorded signals from different elements both within and across different angle transmissions to produce the desired image, thereby taking advantage of the coherence of signals across both elements and transmitted angles.

The CADMAS algorithm comprises the summation of the cross multiplication of the signal across all pairs of elements across all transmitted angles, thereby reinforcing coherent signals and suppressing incoherent noise. This uses a similar principle to DMAS; however, in CADMAS this is extended to all elements across all angular transmissions to take further advantage of the signal coherence across these transmissions. This can be defined by taking the summed cross multiplication of all combinations of different elements across all angles and adding it to the summed cross multiplication of the same elements across different angles. It is worth noting that while expressed in two separate parts this is simply the sum of all pairs of elements over all angles.

(1) $$\begin{array}{l} y_{CADMAS}(x)=\sum_{a=1}^{A}\sum_{b=1}^{A}\sum_{k_{1}=1}^{K-1}\sum_{k_{2}=k_{1}+1}^{K}sign\left(s_{a,k_{1}}\left(\tau_{a,k_{1}}\right)s_{b,k_{2}}\left(\tau_{b,k_{2}}\right)\right)\sqrt{\left|s_{a,k_{1}}\left(\tau_{a,k_{1}}\right)s_{b,k_{2}}\left(\tau_{b,k_{2}}\right)\right|}\\ \;+\sum_{a=1}^{A-1}\sum_{b=a+1}^{A}\sum_{k_{1}=1}^{K}sign\left(s_{a,k_{1}}\left(\tau_{a,k_{1}}\right)s_{b,k_{1}}\left(\tau_{b,k_{1}}\right)\right)\sqrt{\left|s_{a,k_{1}}\left(\tau_{a,k_{1}}\right)s_{b,k_{1}}\left(\tau_{b,k_{1}}\right)\right|} \end{array}$$

where A is the total number of angles, with *a* and *b* referring to summation indexes across angles. K is the total number of transducer elements, with *k*_1_ and *k*_2_ referring to summation indexes across elements. s is the raw signal received at a specific element and transmitted angle, *τ* is the time of flight for a specific transmission angle and element to position *x*, and *y*_*CADMAS*_ is the pixel intensity allocated to position *x*. The schematic for this algorithm can be found in Figure 1. In this form, the CADMAS algorithm, while easily parallelizable, contains a number of multiplications based on the number of cross-angle-element pairings, which can lead to a high computational cost. However, by taking advantage of a mathematical reformulation similar to that described by Polichetti *et al*. [11], If one expands the following summation squared we get:

(2) $$\begin{array}{l} {\left(\sum_{a=1}^{A}\sum_{k=1}^{K}sign\left(s_{a,k}\left(\tau_{a,k}\right)\right)\sqrt{\left|s_{a,k}\left(\tau_{a,k}\right)\right|}\right)}^{2}\\ \;=\left(\sum_{a=1}^{A}\sum_{k=1}^{K}sign{\left(s_{a,k}\left(\tau_{a,k}\right)\right)}^{2}\left|s_{a,k}\left(\tau_{a,k}\right)\right|\right)\\ \;+2(\sum_{a=1}^{A}\sum_{b=1}^{A}\sum_{k_{1}=1}^{K-1}\sum_{k_{2}=k_{1}+1}^{K}sign\left(s_{a,k_{1}}\left(\tau_{a,k_{1}}\right)s_{b,k_{2}}\left(\tau_{b,k_{2}}\right)\right)\sqrt{\left|s_{a,k_{1}}\left(\tau_{a,k_{1}}\right)s_{b,k_{2}}\left(\tau_{b,k_{2}}\right)\right|}\\ \;+\sum_{a=1}^{A-1}\sum_{b=a+1}^{A}\sum_{k_{1}=1}^{K}sign\left(s_{a,k_{1}}\left(\tau_{a,k_{1}}\right)s_{b,k_{1}}\left(\tau_{b,k_{1}}\right)\right)\sqrt{\left|s_{a,k_{1}}\left(\tau_{a,k_{1}}\right)s_{b,k_{1}}\left(\tau_{b,k_{1}}\right)\right|}) \end{array}$$

rearranging this and inserting eqn (1) we get:

(3) $$\begin{array}{l} y_{CADMAS}(x)=({(\sum_{a=1}^{A}\sum_{k=1}^{K}sign(s_{a,k}\left(\tau_{a,k}\right))\sqrt{\left|s_{a,k}\left(\tau_{a,k}\right)\right|})}^{2}\\ \;-\sum_{a=1}^{A}\sum_{k=1}^{K}\mathrm{sign}{(s_{a,k}\left(\tau_{a,k}\right))}^{2}\left|s_{a,k}\left(\tau_{a,k}\right)\right|)/2 \end{array}$$

This formulation brings the computation time down to 1–2 times that of DAS. This is due to the fact that the algorithm can be implemented by performing a modified form of DAS twice, once with the magnitudes square-rooted and the final beamformed values squared, and a second time with the phase information squared, before finally subtracting one from the other. This would give a computation time roughly equivalent to twice that of DAS. However, as these two calculations do not need to be performed sequentially, depending on the implementation and hardware these two operations could be performed in parallel. This would bring the computation time down to be similar to that of DAS. However, this would require sufficient hardware to provide twice the number of parallel computational streams, with anything done to improve the computational cost of DAS (including parallelisation), propagating through to improvements in CADMAS’s computation speed. It is worth noting that, in theory, if applied one angle at a time and summing the final values, DMAS could also be implemented with this mathematical reformulation, thereby reducing its computation time as well.

#### Experimental data acquisitions and evaluation

To investigate the effectiveness of the CADMAS algorithm, we conducted a series of experiments in which CADMAS was compared to DAS, FMAS, and DMAS, in addition to DMAS+FMAS, in which DMAS beam-forming is implemented for each angle and then the angles are compounded with the FMAS algorithm. It is worth noting that in the case of both DAS and DMAS conventional angular compounding was performed via summation. Across all of the algorithms, the receive sensitivity of the element was applied to each pixel location [3,12], ensuring that only correlating signals were applied to each pixel, envelope detection was conducted at the end for all algorithms using the traditional Hilbert transform method. Initially, we studied the effect of CADMAS on the point spread function of a wire scatterer. We imaged a 50 *μ*m wire at a distance of 26 mm from a GE L3-12-D linear array transducer operated with a Vantage 256 Verasonics scanner with a transmitted frequency of 5 MHz, 0.1 MI, 0.5 cycle, 25% Tukey apodization on transmit, and 10 transmit angles over a ± 10° range using triangular transmission [13]. In this and all future experiments data was sampled at four samples per wavelength using the arrays central frequency, collected as real data.

Following this in order to quantify the improvement in image contrast in a controlled in vitro environment, we utilized a GAMMEX phantom (GAMMEX Ultrasound 403 LE multipurpose phantom) using a GE L3-12-D array with a transmitted frequency of 5 MHz, 0.3 MI, 1.5 cycles, 25 % Tukey apodization on transmit, 11 transmit angles over a ± 10° range using triangular transmission [13]. We then calculated Contrast-to-Noise Ratios (CNRs) to allow for quantitative comparisons, according to:

(4) $$CNR=20log10\left(\frac{\mu_{T}-\mu_{v}}{\sigma_{v}}\right)$$

The contrast ration (CR) according to:

(5) $$CR=20log10\left(\frac{\mu_{T}}{\mu_{v}}\right)$$

And the speckle SNR is according to:

(6) $$speckleSNR=20log10\left(\frac{\mu_{T}}{\sigma_{v}}\right)$$

where *μ*_*T*_ is the mean of the pixel intensities in the tissue, *σ*_*v*_ is the standard deviation of the pixel intensities in the tissue. *μ*_*v*_ is the mean of the pixel intensities in the void and *σ*_*v*_ is the standard deviation of the pixel intensities in the void. We additionally calculated the CNR for images generated using the partial histogram matching technique described by Bottenus et al. [14], and additionally quantified using the gCNR technique [15]. The CNR and CR were chosen as they are both commonly used metrics that quantify the levels of contrast generated. Speckle SNR was chosen as it is indicative of the sharpness of the image [16]. gCNR has become a standard method of comparing image contrasts that is more robust to S shaped shifts in the dynamic range compared to more conventional CNR and CR [15]. Histogram matching was performed as it is a technique that has recently shown promise in being more generally robust to nonlinear dynamic range shifts than CNR and CR [14].

To test the CADMAS algorithm for use with amplitude modulation (AM), a contrast-specific mode initially designed to image nonlinear scatters. A 2 mm diameter cylinder of gas vesicles (GVs) set inside a tissue mimicking phantom comprising 1% (w/v) agar (Agar W201201, SigmaAldrich) and 0.2 % (w/v) Al_2_O_3_ (White Aluminium Oxide Powder Fepa Grit: 1200, 3 Micron, Kemet) was imaged using an L11-4v (Verasonics). With a transmitted frequency of 4 MHz, 1 cycle, pressure ranging from 120 to 540 kPa peak positive pressure, 25 % Tukey anodization on transmit, and 11 transmit angles over a ± 10° range using triangular transmission [13]. For CNR calculations the region containing the GVs was used in place of the “tissue” in eqns (4) and (5) with a region in the tissue mimicking the phantom taking the place of the “void”. GVs are protein-shelled, air-filled sub-micron particles [17] naturally expressed by buoyant photosynthetic microbes [18]. They can be purified as nano-scale contrast agents or expressed heterologously in bacterial and mammalian cells as acoustic reporter genes [19–21]. GVs provide strong contrast to tissue using AM imaging [22,23]. AM images are created by sending out three transmissions for each angle, the second of which is conducted at the standard (full) pressure intensity, and the first and third pulses are transmitted at half the pressure intensity, this is usually referred to as a half-full-half transmission technique, the received signals from these three transmissions are then summated prior to beamforming. We acquired images of GVs purified from *Anabaena flos-aquae* [24,25] and analyzed the CNR at each transmitting pressure.

### In vivo evaluation

The ability of CADMAS to improve *in vivo* images was investigated by analyzing images of a rabbit kidney. The experiment complied with the Animals (Scientific Procedures) Act 1986 and received approval from the Animal Welfare and Ethical Review Body of Imperial College London. A pathogen-free male New Zealand White rabbit (HSDIF strain, Envigo, Huntingdon, UK) aged 20 wk and weighing 3.14 kg, it was maintained on a 12:12 h light:dark cycle at 188C and fed a standard laboratory diet. It was pre-medicated with acepromazine (0.5 mg/kg, i.m., CVet, London, UK), anesthetized using a combination of medetomidine (Domitor, 0.25 mL/kg, i.m.) and ketamine (Narketan, 0.15 mL/kg, i.m.) and maintained with a third of the initial dose of anesthesia every 30 to 45 min for up to 4 h. The rabbit underwent tracheotomy and was ventilated at 40 breaths/min. It was positioned supine on a heated mat with, continuous monitoring of heart rate and blood oxygen saturation, and fur was removed from the abdominal region. The left kidney was imaged transcutaneously after the injection of microbubble contrast agents via the marginal ear vein; an initial dose of 0.1 mL was followed by a second of 0.05 mL. The perfluorobutane microbubbles were prepared in-house by dissolving 1,2-Dipalmitoyl-sn-glycero-3-phosphatidylcholine (Avanti, Merck, MO, USA), 1,2-dipalmitoyl-sn-glycero-3-phosphatidyleth-anol-amine-polyethylene glycol 2000 (Avanti), and 1,2-dipalmitoyl-3-trime-thylammonium propane (Avanti), in water with a molar ratio of 65:5:30 and a total lipid concentration of 0.75, 1.5 and 3 mg/mL respectively. This resulted in a solution of 15% propylene, 5% glycerol and 80% saline. Perfluorobutane (F2 Chemicals Ltd, Preston, UK) was injected into a 2 mL container containing 1.5 ml of the prepared solution just before use and the microbubbles, were activated by agitation in a shaker for 60 s, giving a final concentration of 5 × 10^9^ microbubbles/mL [26]. Euthanasia employed pentobarbital (0.8 mL/kg). We imaged the kidney transcutaneously using a GE L3-12-D probe with a transmitted frequency of 5 MHz, 0.1 MI, 1 cycle, 100 frames per second, and 10 angles ± 10°. Images were then accumulated from 500 sequential frames. For CNR and CR calculations the region shown in blue (Fig. 5) was used in place of the “tissue” in eqns (4) and (5) with the region shown in blue taking the place of the “void”.

## Results

**Figure 2:**
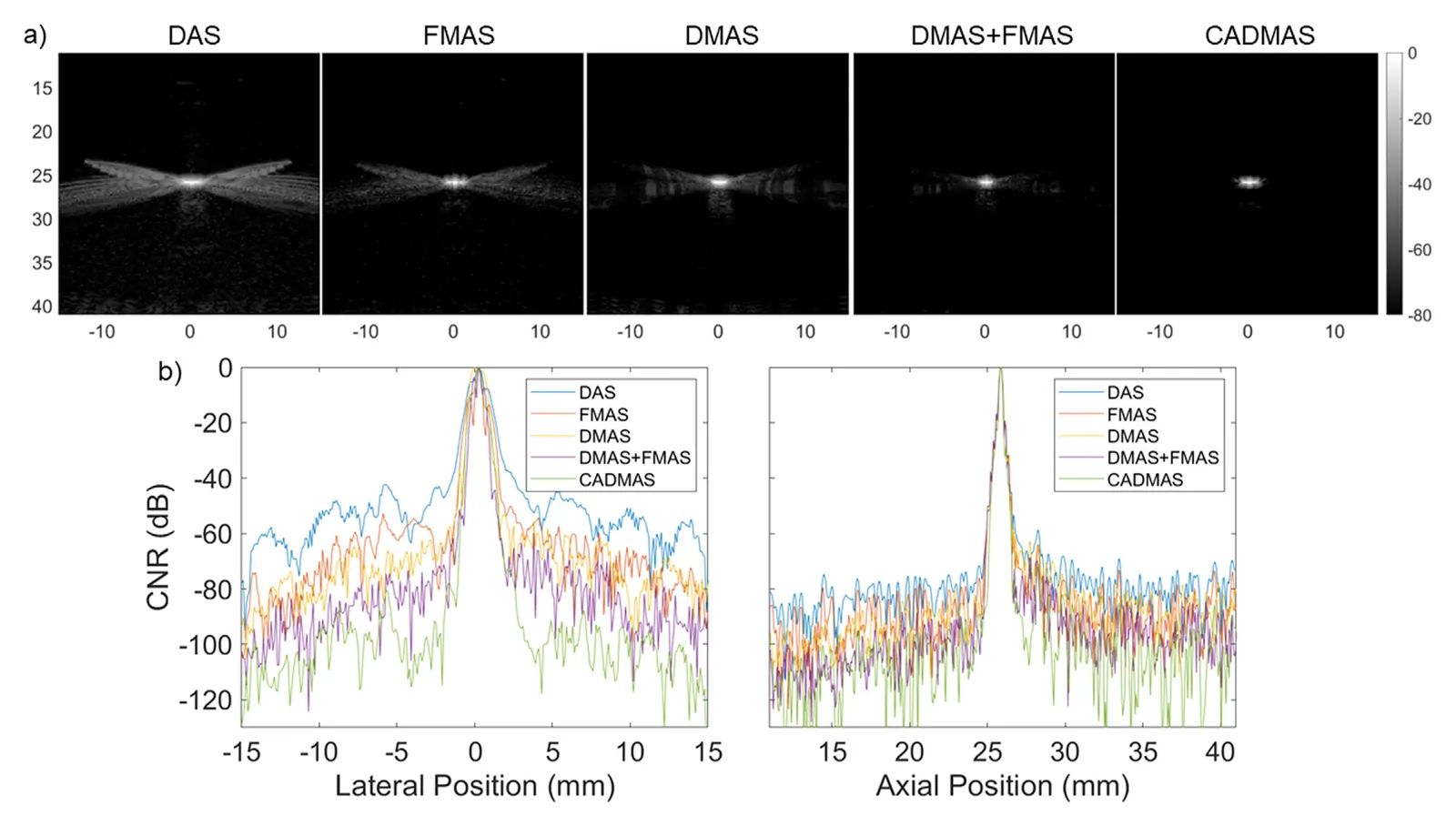
(a) Images obtained by beamforming an experimental wire scatter dataset in five different ways. (b) Lateral and axial cross sections of the images for comparison.

In our wire scatterer dataset looking at the point spread function we found that the point spread function of CADMAS had roughly 40 dB reduction in side-lobes and noise floor compared to DAS and roughly 20 dB reduction over DMAS (Fig. 2). To show some example computation times, for this dataset the computation times per frame were: DAS: 0.054 s, FMAS: 0.056 s, DMAS: 1.5 s, FMAS+DMAS: 1.5 s, and CADMAS: 0.057 ss. Showing that CADMAS only marginally increases the computation time. These times were calculated by taking the average computation time over 100 frames, with all algorithms implemented on a GPU with parallelization.

**Figure 3:**
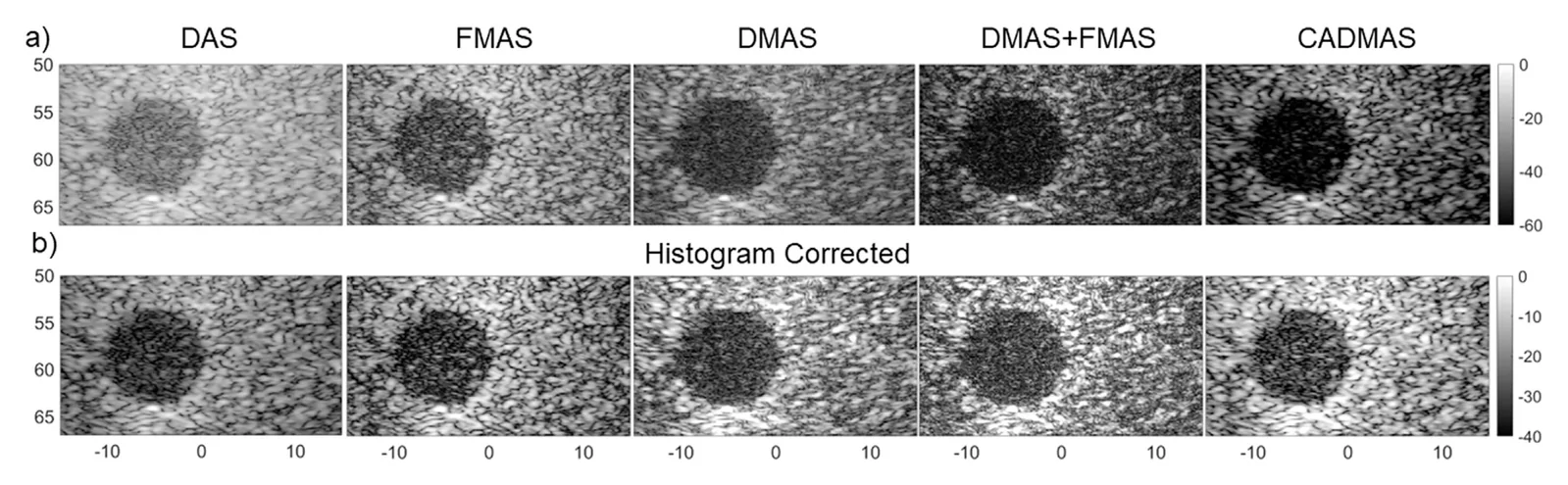
(a) Images obtained by beamforming a GAMEX phantom void dataset in five different ways. (b) The same images after histogram correction.

For the GAMMEX Phantom dataset the images produced by the five algorithms can be seen in Figure 3a where the contrast enhancement of CADMAS can be seen. The images generated following histogram correction can be seen in Figure 3b, where the contrast enhancement of CADMAS, while reduced, is still present. CNR, CR, and gCNR values are shown in Table 1. Overall a 7.6 dB improvement in CNR and 11.8 dB improvement in CR was found for CADMAS over DAS, with a 4.8 dB and 9.1 dB improvement over DMAS respectively. Post histogram correction these improvements became 3.4 dB (CNR) and 5.4 dB (CR) for CADMAS over DAS, with 0.7 dB (CNR) and 2.8 dB (CR) over DMAS. The interpretation of the relative histogram corrected dB values is complicated due to the scaling involved. However, combined with the gCNR values, the results suggest that a portion, but not all of, the apparent improvement for all of these non-linear beamformers is due to a shift in the dynamic range, and that the contrast improvement of CADMAS over DMAS is between 26% and 108% of the improvement of DMAS over DAS depending on which metrics are chosen. The average improvement was 72% across the three comparison metrics. Speckle SNR was also calculated both pre and post histogram correction, with the results suggesting that all of the non-linear techniques provide increased sharpness over DAS, hence the decrease in speckle SNR, with DMAS+FMAS providing the sharpest images and FMAS, DMAS, and CADMAS delivering comparable sharpness, with perhaps a slight decrease in the case of FMAS.

**Figure 4:**
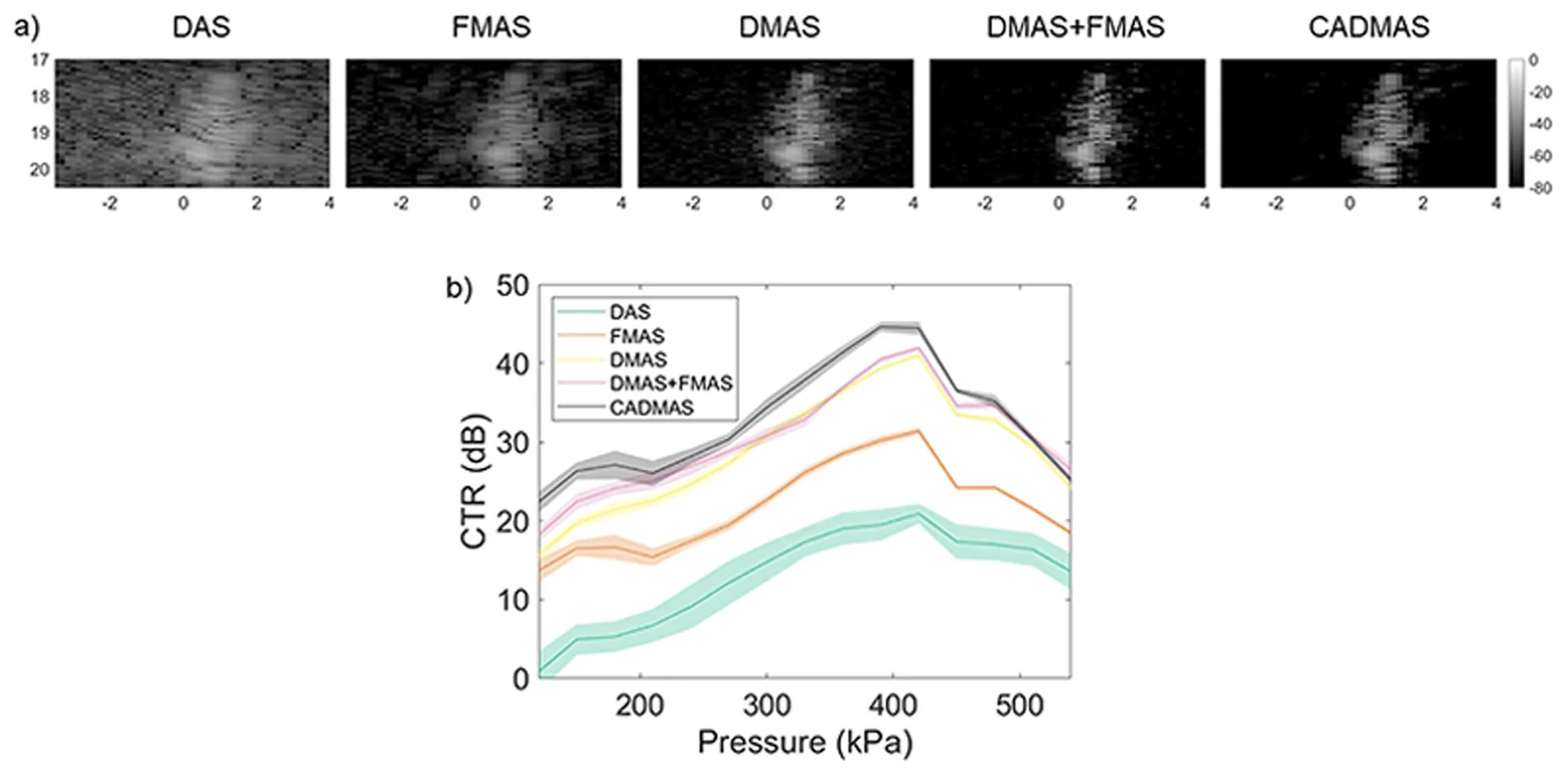
(a) AM images obtained by beamforming an imaging gas vesicles embedded in a tissue phantom at 430 kPa peak negative pressure in five different ways. (b) Mean and standard deviation of the CNR calculated for each beamforming technique throughout the applied pressure ramp (n=10).

When we look at our *in vitro* AM dataset using GVs, we can see a representative image at 420 kPa in Figure 4a and the relationship between the transmit pressure and the CNR in Figure 4b. Roughly a 20 dB improvement in CNR was found in CADMAS over DAS, with a roughly 5 dB improvement over DMAS.

**Figure 5:**
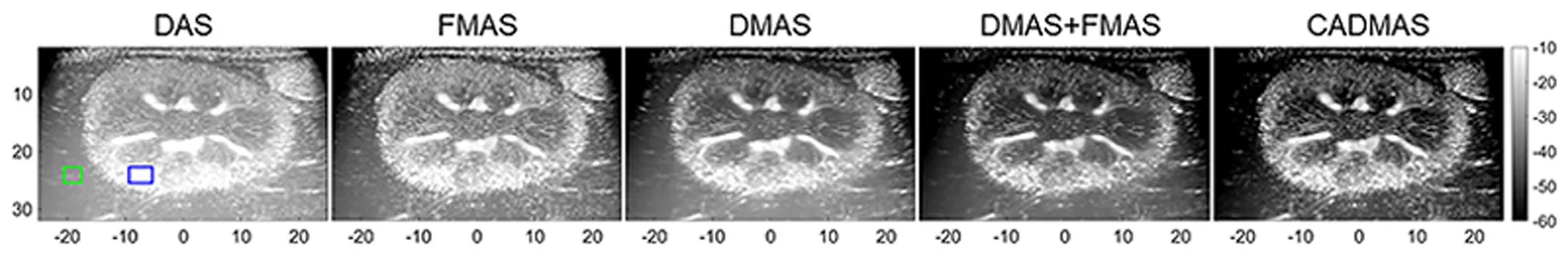
Images accumulated over 500 frames by beamforming a rabbit kidney dataset in five different ways, Contrast and tissue regions used for CNR estimations are designated by blue and green boxes, respectively.

The *in vivo* rabbit kidney data can be seen in (Fig. 5), the CADMAS beamformer enhanced contrast while suppressing incoherent signals and yielded the highest CNR, CR, and gCNR across the five imaging methods (Table 2). A 12.2 dB improvement in CNR was found by CADMAS over DAS, with a 6.3 dB improvement over DMAS.

## Discussion

The results of the wire scattering experiment clearly demonstrate the ability of CADMAS to suppress side lobes and improve contrast. These results follow through and can be seen qualitatively across all experiments. As both qualitatively and quantitatively contrast is consistently enhanced across all experiments and all contrast metrics for CADMAS over DAS, DMAS, FMAS, and DMAS+FMAS. This is achieved with a computation time that should be between 1 and 2 times that of DAS. In our implementation we experienced a less than 6% increase in computation time. This taken together demonstrates the potential of the algorithm to be used by both researchers and clinical systems.

It should be noted that a quantitative comparison of images with different beamforming methods is difficult to conduct fairly. Many metrics and methods have been derived, each having strengths and weaknesses [14,15,27]. A key issue is the effect of nonlinear shifts in the dynamic range, which can artefactually affect metrics such as the commonly used CNR and CR. In the absence of a single flawless metric and in an attempt to conduct a fair comparison, we calculated several comparison metrics. While each metric individually has limitations, agreement between them is indicative of true contrast improvement, as each is resistant to different types of dynamic range shifting and has been used previously to evaluate beamforming methods. As such, the CNR and CR for both our original images (Fig. 3a) and for images generated using the partial histogram matching technique described by [14] (Fig. 3b), and additionally quantified using the gCNR technique [15]. CNR, CR, and gCNR values were higher for CADMAS compared to other beamformers regardless of scaling.

In the GV experiments, as expected from the nonlinear mechanical behavior of GVs, CNR increased with pressure until a pressure was reached at which the GVs started to collapse irreversibly and the CNR began to decrease. This matches the results for GVs that have been found previously [28].

## Conclusions

In this study we show that the proposed CADMAS algorithm provides greater contrast than DAS, DMAS, FMAS, and DMAS+FMAS, and at a low computational cost of only 6 % slower than DAS. The improvements were demonstrated in both conventional B-Mode and AM imaging, with GV and microbubble contrast agents, and for *in vitro* and *in vivo* imaging. The results suggest that CADMAS has potential utility in biological research and clinical settings.

## Supplementary Material

Supplementary material associated with this article can be found in the online version at doi:10.1016/j.ultrasmedbio.2025.05.023.

Supplementary Materials

## Figures and Tables

**Table 1:** Mean and standard deviation of contrast-to-noise ratio, contrast ratio, gCNR, and speckle SNR over multiple acquisitions when beamforming GAMMEX void phantom datasets in five different ways (n = 10 acquisitions)

	*DAS*	*FMAS*	*DMAS*	*DMAS* + *FMAS*	*CADMAS*
CNR	16.0 ± 0.2	16.2 ± 0.4	18.8 ± 0.5	16.8 ± 0.6	23.6 ± 0.7
CR	12.9 ± 0.3	16.9 ± 0.4	15.6 ± 0.3	16.9 ± 0.4	24.7 ± 0.5
Speckle SNR	6.1 ± 0.1	2.6 ± 0.1	1.8 ± 0.2	−1.4 ± 0.3	0.5 ± 0.2
gCNR	0.803 ± 0.008	0.889 ± 0.007	0.897 ± 0.006	0.924 ± 0.006	0.975 ± 0.003
CNR (Histogram Corrected)	16.0 ± 0.2	14.9 ± 0.4	18.7 ± 0.5	15.5 ± 0.5	19.4 ± 0.5
CR (Histogram Corrected)	12.9 ± 0.3	14.5 ± 0.3	15.5 ± 0.3	14.9 ± 0.4	18.3 ± 0.4

**Table 2:** Contrast-to-noise ratio, contrast ratio, and gCNR when beam-forming an *in vivo* rabbit kidney dataset in five different ways

	*DAS*	*FMAS*	*DMAS*	*DMAS* + *FMAS*	*CADMAS*
CNR	33.9	37.7	39.8	44.3	46.1
CR	15.8	19.7	20.8	22.2	28.3
gCNR	0.89	0.95	0.96	0.97	0.99

## Data Availability

Data are available from the corresponding author upon reasonable request.
